# Cas12a and MAD7, genome editing tools for breeding

**DOI:** 10.1270/jsbbs.23049

**Published:** 2024-02-22

**Authors:** Shunya Hozumi, Yi-Chen Chen, Tatsuya Takemoto, Shun Sawatsubashi

**Affiliations:** 1 Setsuro Tech Inc., Fujii Memorial Institute of Medical Sciences, 3-18-15 Kuramoto-cho, Tokushima 770-8503, Japan; 2 Laboratory for Embryology, Institute for Advanced Medical Sciences, Tokushima University, 3-18-15 Kuramoto-cho, Tokushima 770-8503, Japan; 3 Research and Innovation Liaison Office, Institute for Advanced Medical Sciences, Tokushima University, 3-18-15 Kuramoto-cho, Tokushima 770-8503, Japan

**Keywords:** genome editing, CRISPR, Cas12a, MAD7, ST8, breeding

## Abstract

Food shortages due to population growth and climate change are expected to occur in the near future as a problem that urgently requires solutions. Conventional breeding techniques, notably crossbreeding and mutation breeding, are known for being inefficient and time-consuming in obtaining seeds and seedlings with desired traits. Thus, there is an urgent need for novel methods for efficient plant breeding. Breeding by genome editing is receiving substantial attention because it can efficiently modify the target gene to obtain desired traits compared with conventional methods. Among the programmable sequence-specific nucleases that have been developed for genome editing, CRISPR–Cas12a and CRISPR–MAD7 nucleases are becoming more broadly adopted for the application of genome editing in grains, vegetables and fruits. Additionally, ST8, an improved variant of MAD7, has been developed to enhance genome editing efficiency and has potential for application to breeding of crops.

## Introduction

The global population is expected to reach 9.6 billion by 2050, the feeding of which would require a 60% increase in crop production yields (Chen *et al.* 2019, Gao 2021). To successfully feed this rapidly growing population in the face of climate change and decreased arable land, there is an urgent need for innovations in crop breeding technologies to increase productivity and accelerate sustainable agricultural development. Crossbreeding, mutation breeding, and transgenic breeding are currently the primary methodologies for plant improvement in modern agriculture. However, the acquisition of desirable alleles through crossbreeding is a time-consuming process. Mutation breeding, wherein random mutations are induced through chemical or physical irradiation, proves beneficial in acquiring desirable traits. Nevertheless, this methodology is economically demanding due to the stochastic nature of random mutations, necessitating extensive screening on a large scale. For these reasons, there is a real possibility that crossbreeding and mutation breeding may not be able to meet the increasing demands for crop production in the future. Transgenic breeding, which involves the transfer of exogenous genes to obtain desirable traits, is useful for plant enhancement for the augmentation of production. However, the commercialization of these genetically modified organisms (GMOs) is limited by long and costly regulatory evaluation processes (Chen *et al.* 2019). Genome editing techniques can address the challenges associated with conventional plant breeding methods, as they enable precise and predictable modifications to the genome for obtaining desired traits. This suggests a potential for genome editing to be a cost-effective approach compared to conventional plant breeding methods.

Genome editing is carried out using programmable sequence-specific nucleases, including meganucleases (MegNs), zinc finger nucleases (ZFNs), transcription activator-like effector nucleases (TALENs), and clustered regularly interspaced short palindromic repeats (CRISPR)/CRISPR-associated protein (CRISPR–Cas) (Doudna and Charpentier 2014, Hillary and Ceasar 2023, Hsu *et al.* 2014, Khalil 2020, Sun and Zhao 2013, Urnov *et al.* 2010). These nucleases induce double-strand breaks (DSBs) at target sites in DNA and generate precision genome modifications through DNA repair pathways. Genome-edited products are entering the market. For example, GABA-enriched tomatoes produced by genome editing technology are commercially available in Japan (Waltz 2022). Moreover, in the United States, the oleic acid-enriched soybean generated by genome editing is on the market (Liu *et al.* 2021), and the cattle with a mutation by CRISPR technology to have short and slick coats that let them better withstand hot weather, are under review by the FDA (Harrison 2022). While meganucleases, ZFNs, and TALENs recognize target sequences via protein–DNA interactions, the CRISPR–Cas system employs guide RNA to target DNA sequences. The CRISPR–Cas system has emerged as the most widely used option for genome editing in agriculture, owing to its cost-effectiveness, simplicity, and high efficiency. Among the various CRISPR systems, Cas9 has been the most widely used and is considered the most advanced in terms of its applications. An abundance of reports have already been published on the use of the CRISPR–Cas9 system to produce genome-edited grains, vegetables, and fruit, among others (Ahmad 2023, Devi *et al.* 2022, Gan and Ling 2022, Guo *et al.* 2023, Liu *et al.* 2022, Wang *et al.* 2022). Consequently, this review is centered on CRISPR–Cas12a, a unique effector distinct from CRISPR–Cas9; CRISPR–MAD7, a Cas12a variant originating from *Eubacterium rectale*; and ST8, an improved variant of MAD7.

## Genome editing with Cas12a in crops

While the CRISPR–Cas9 system is the most popular tool for plant genome editing, as described above, the CRISPR–Cas12a nuclease is becoming more broadly adopted for genome editing applications in various organisms, including plants, bacteria, fungi, human cell lines, and animals. Notable plants undergoing genome editing with CRISPR–Cas12a include rice, maize, wheat, tomato, Duncan grapefruit, apple, cotton, and tobacco (Table 1).

Numerous reports have documented the use of the CRISPR–Cas12a system in rice. It was reported that codon-optimized FnCas12a generated indel mutations in *OsDL* and *OsALS* genes at an average frequency of 47.2% in rice (Endo *et al.* 2016). The upregulated by transcription activator-like 1 (UPT) effector box in the promoter region of the rice *OsXa13* gene plays a key role in the pathogenicity of *Xanthomonas oryzae* pv. oryzae (Xoo), which causes rice leaf blight, a devastating global disease. The mutation rates of *OsXa13* promoter targeted by two LbCas12a vectors were 39.6% and 56.4%. The majority of the deletions among the mutations ranged from 8 to 10 bp in size. Core nucleotide deletion in the UPT box of the *OsXa13* promoter conferred resistance to rice blight (Yu *et al.* 2021). Both FnCas12a and LbCas12a have demonstrated the ability to achieve targeted gene insertions via HDR into the *Chlorophyllidea oxygenase* (*CAO1*) gene, which converts chlorophyll a to chlorophyll b. The CRISPR–Cas12a vector and donor template plasmid were co-transformed into rice embryogenic calli using the bombardment method. Disruption of the *OsCAO1* gene results in a yellow leaf phenotype, which was used as a visual marker for targeted gene insertion. FnCas12a exhibited a higher frequency of targeted insertion than LbCas12a (Begemann *et al.* 2017). LbCas12a was also used for targeted gene replacement with donor DNA harboring point mutations in the *OsALS* gene in rice (Li *et al.* 2018). Both FnCpf1 and LbCpf1 generated mutations simultaneously at all four target sites within the *OsRLK* and *OsBEL* genes at mutation frequencies of 34.4% and 40.0% respectively, using a plasmid harboring multiple guide RNA expression cassettes in rice (Wang *et al.* 2017). Genome editing in rice protoplasts and plants was successfully achieved with vectors harboring a single transcript unit expressing LbCas12a and a single crRNA or four crRNAs under the control of the Pol II promoter (STU–Cas12a system). The system is based on self-processing of the crRNA array by Cas12a. Editing efficiencies ranging from 29.2% to 50% were observed at the four target sites in *OsDEP1* and *OsROC5* genes with this system (Tang *et al.* 2019). In the STU–Cas12a system, the mutation frequency in *OsPDS* and *OsGS3* genes induced by a vector harboring poly-A at the 3ʹ-end of LbCas12a cDNA was 1.9-fold higher on average than that obtained with a vector lacking poly-A in rice (Xu *et al.* 2019). The influence of the crRNA structure in the genome editing of rice with Cas12a has also been studied. LbCas12a induced indel mutations at *OsPDS* and *OsBEL*, with mutation frequencies of 21.4% and 41.2%, respectively, using longer pre-crRNAs with the full-length repeat-spacer-repeat sequence (Xu *et al.* 2017). FnCas12a-mediated genome editing efficiency at each target site varies depending on the length of the crRNA guide sequence in rice calli. For example, higher mutation frequencies were observed at target sites of *OsAAO2* and *OsALS* when using crRNA with an 18-nt guide sequence and a 30-nt guide sequence, respectively (Negishi *et al.* 2020). To achieve high editing efficiency with Cas12a systems in rice, high-temperature regimes have been explored. Nearly 100% mutations were detected in T0 rice plants targeting *OsDEP1* and *OsROC5* using AsCas12a by co-culturing with *Agrobacterium* at 25°C, selecting at 32°C, and regenerating shoots at 28°C (Malzahn *et al.* 2019).

Cas12a is also applicable to a wide range of crops besides rice. In wheat, LbCas12a has successfully induced indel mutations in the *GUS* gene at an editing efficiency of 3.1% (Liu *et al.* 2020a). In addition, in maize, the editing efficiency of *ZmO2* in Cas12a-edited T1 ranged from 0% to nearly 50%. The editing efficiency of Cas12a was found to be positively correlated with its expression level (Gong *et al.* 2021). High-temperature regimes have been explored to achieve high editing efficiency with Cas12a systems in maize. High mutagenesis frequencies of *ZmGL2* were obtained in the T1 generation from T0 lines expressing LbCas12a with a temperature setting of 28°C/21°C (day/night) in maize (Malzahn *et al.* 2019). In tomato, gene disruption with targeted insertion at the *SlAnt1* gene and targeted gene replacement of salt tolerance allele of *SlHKT1;2* genes were achieved using a geminivirus replicon system, demonstrating high efficiency of homology-directed repair (van Vu *et al.* 2020). In apple, LbCas12a successfully generated deletions in two different exons of the *MdPDS* gene, resulting in an albino phenotype. The deletion size at one locus ranged from 1 to 84 bp, with an average of 12.4 bp, while at the other locus, the deletion sizes ranged from 2 to 38 bp, with an average of 13.0 bp (Schröpfer and Flachowsky 2021). In Duncan grapefruit, LbCas12a induced indel mutations in *CsPDS* and type I and type II *CsLOBP*s (Jia *et al.* 2019). Moreover, in cotton, LbCas12a demonstrated high gene editing efficiency, with 87% of T0 plants carrying indel mutations in the *GhCLA1* gene (Li *et al.* 2019). Furthermore, in tobacco, codon-optimized FnCas12a resulted in indel mutations, with an average frequency of 28.2% in the *NtPDS* and *NtSTF1* genes (Endo *et al.* 2016).

In a comparative study between Cas12a and Cas9, *OsEPFL9* heterozygous mutated T0 plants generated using LbCas12a were present at a higher frequency (10%) than for those with Cas9 (4%) in rice (Yin *et al.* 2017). When the bombardment method was employed to deliver two Cas9 (wild-type Cas9 and high-fidelity Cas9) and two Cas12a (LbCas12a and AsCas12a) to induce mutations in the *OsPDS* gene, the mutation frequency of LbCas12a (32.3%) surpassed those of wild-type Cas9 (3.6%) and high-fidelity Cas9 (8.8%) in rice (Banakar *et al.* 2020). Conversely, in maize, 90% to 100% of the Cas9-edited T0 plants carried indel mutations in the *ZmGL2* gene, whereas 0% to 60% of LbCas12a-edited T0 plants did so (Lee *et al.* 2019b).

In a comparative study between LbCas12a and FnCas12a, LbCas12a exhibited higher editing efficiency than FnCas12a at all of the target sites within the *OsEPSPS*, *OsBEL*, and *OsPDS* genes in rice (Wang *et al.* 2017). The observed mutation frequencies ranged from 0.6% to 10% with AsCas12a and from 15% to 25% with LbCas12a across six targets of *OsPDS*, *OsDEPI*, and *OsROC5* genes in rice. More than 90% of the mutations induced by both AsCas12a and LbCas12a were deletions, with the majority ranging in size from 6 to 13 bp (Tang *et al.* 2017).

Various Cas12a variants have been reported in the context of genome editing of crops. In rice, FnCpf1 was demonstrated to exert activity against canonical TTTV PAM sequences and a TTV PAM site with VTTV PAM combinations, while it did not exhibit activity against GTTA and GTTC PAM sites (Zhong *et al.* 2018). Notably, a high success rate was achieved in rice protoplast for editing CCCC and TYCV PAM sites using the LbCpf1-G532R/K595R (RR) variant and TATG PAM sites using the LbCpf1-G532R/K538V/Y542R (RVR) variant (Zhong *et al.* 2018). Additionally, the LbCas12a-G146R/D156R/R182V (RRV) variants demonstrated the generation of indel mutants in *OsGA1*, *Os11g20160*, *Os01g09810*, and *Os11g19880* with nearly 100% editing efficiency in T0 plants. Remarkably, this highly efficient editing with LbCas12a-RRV was observed at the non-canonical VTTV PAM sites (Zhang *et al.* 2023).

The major differences between Cas9 and Cas12a proteins include the following (Bandyopadhyay *et al.* 2020, Senthilnathan *et al.* 2023):

(1) Cas9 is a class II type II endonuclease, which contains two different nuclease domains, HNH and RuvC, whereas Cas12a is a class II type V endonuclease, which contains a RuvC-like endonuclease domain with a Nuc domain for the cleavage of target and nontarget DNA strands.

(2) The protospacer adjacent motif (PAM) sequence of Cas12a is “TTTN”, which is suitable for targeting T-rich regions of the genome, in contrast to the G-rich PAM favored by Cas9.

(3) Cas12a cleaves the target DNA strand 18–23 nucleotides (nt) distal to the PAM, producing staggered ends, whereas Cas9 generates blunt ends 3 nt upstream of the PAM site.

(4) Cas12a requires only a single crRNA, which reduces the complexity of the editing system, whereas Cas9 requires tracrRNA and crRNA.

(5) As opposed to Cas9, Cas12a possesses intrinsic RNase activity to process its own crRNA array, making it an excellent platform for multiplexed editing.

(6) Cas12a has the potential to decrease off-target effects due to its PAM (TTTN) compared with the Cas9 PAM (NGG). It has been reported that off-target mutations were not detected in two-mismatch and three-mismatch off-target sites in plants (Modrzejewski *et al.* 2020).

(7) Compared with Cas9, the Cas12a orthologs in use are not effective at lower temperatures. Although genome editing was performed by LbCas12a in *Arabidopsis* plants grown at 22°C using a vector harboring promoters applied for Cas9 genome editing with high efficiency, mutations were not detected in the plants (Malzahn *et al.* 2019).

(8) Cas12a exhibits nonspecific collateral ssDNA cleavage activities (trans-cleavage) after being activated by the specific recognition of target nucleic acids.

## CRISPR–MAD7 system

CRISPR–Cas12a nuclease MAD7, which was released by Inscripta Inc., is an engineered Cas12a variant originating from *Eubacterium rectale* found on the island of Madagascar. It shares 76% identical nucleotides with the native form (Liu *et al.* 2020b, Rojek *et al.* 2023, Vanegas *et al.* 2023). It encodes a monomeric 147.9 kDa polypeptide consisting of 1,263 amino acids. Notably, MAD7 shares just 31% amino acid homology with the canonical *Acidaminococcus* Cas12a (also known as AsCpf1) (Lin *et al.* 2021, Liu *et al.* 2020b, Price *et al.* 2020, Rojek *et al.* 2023).

MAD7 shows a preference for 5ʹ-YTTN-3ʹ PAM sites, making it available for genome editing in T-rich DNA sequences, whereas PAM sites of Cas9 are in the form 5ʹ-NGG-3ʹ, making it useful in G-rich DNA sequences (Mund *et al.* 2023, Price *et al.* 2020, Rojek *et al.* 2023). Mad7 crRNAs are designed with 5ʹ direct repeats of either 21 or 35 nucleotides, followed by a 21-nucleotide protospacer region at the 3ʹ end. Like other Cas12a, MAD7 does not require trans-activating CRISPR RNA (tracrRNA), which plays a role in the maturation of crRNA in the CRISPR–Cas9 system (Rojek *et al.* 2023). Meanwhile, like other CRISPR–Cas12a systems, MAD7 generates a cohesive end at the 5ʹ end of crRNA after cleavage of the double strand, whereas Cas9 generates a blunt end three bases upstream of the PAM site (Rojek *et al.* 2023, Vanegas *et al.* 2023).

### MAD7 is available in a broad range of species

MAD7 has been used in genome editing of various cell lines and species (Table 2) (Lin *et al.* 2021, Liu *et al.* 2020b, Mund *et al.* 2023, Price *et al.* 2020, Rojek *et al.* 2023, Vanegas *et al.* 2023, Wierson *et al.* 2019). Although only one report has been published on the genome editing of plants, in contrast to the case for animals, MAD7 is available for a high-fidelity system and generates indel mutations with similar efficiency to CRISPR–LbCas12a. MAD7 is useful for multiplex gene editing using crRNA expression vectors that include ribozyme sequences in the protoplast (Lin *et al.* 2021). Furthermore, mutant rice plants were obtained at 49.0%–65.6% efficiency in *OsALS*, *OsEPSPS*, and *OsNRAMP5* genes with no off-target events using MAD7 by *Agrobacterium*-mediated transformation. By particle bombardment, transgene-free *TaDEP1* and *TaVRN1* mutants of wheat plants in the T_0_ generation was generated at editing frequencies of 1.5% and 3.0%, respectively (Lin *et al.* 2021). Moreover, MAD7 with APOBEC3A deaminase and UDG (Uracil-DNA Glycosylase) was shown to be useful for generating predictable deletions from 5ʹ-deaminated cytosines to the MAD7 cleavage site in protoplasts as AFIDs (APOBEC–Cas9 fusion-induced deletion systems) (Lin *et al.* 2021, Wang *et al.* 2020).

MAD7 is also available for genome editing in four different aspergilli that have not previously been genetically engineered (Vanegas *et al.* 2023). In aspergilli, mutations were successfully generated in non-homologous end-joining (NHEJ)-deficient strains and NHEJ-proficient strains by genome editing with MAD7. Mutations were induced by introducing a stop codon with donor ssDNA or by gene disruption with knock-in of an mRFP fragment through the homologous recombination pathway in non-homologous end-joining (NHEJ)-deficient strains, including *Aspergillus nidulans* strain NID1 and *A. niger* strain NIG96. Meanwhile, gene disruptions were induced by indel or knock-in of a DNA fragment through the NHEJ pathway in NHEJ-proficient strains, including *A. niger* strain NIG1, *A. oryzae* strain ORY2, and *A. campestris* wild-type strain (Vanegas *et al.* 2023).

In bacteria, it was also reported that *Bacillus subtilis* and *Escherichia coli* underwent genome editing using MAD7 (Mund *et al.* 2023, Price *et al.* 2020). MAD7 induced mutation at a high rate with donor DNA with a 1 kbp homology arm through homologous recombination machinery, the same as Cas9 in *Bacillus subtilis* (Price *et al.* 2020). Additionally, catalytically inactive MAD7 (dMAD7) variants (D877A, E962A, and D1213A) identified as having sequence homology with the catalytic residues of AsCpf1 were shown to be useful for CRISPR interference (CRISPRi). dMAD7 was targeted to the 5ʹ end of target genes of the *amyE* and *gfpmut3* genes, and the expression of these genes was downregulated by up to 71.3% at single and multiplexed target sites within *B. subtilis* (Price *et al.* 2020). In *Escherichia coli*, MAD7 is available as a genome editing tool in combination with a λ-Red recombination system, which was reported to have successfully achieved sequential genome editing of multiple loci (Datsenko and Wanner 2000, Mund *et al.* 2023). The λ-Red recombination system functions in the homologous recombination of donor DNA following genome editing by MAD7 (Mund *et al.* 2023).

MAD7 has also been successfully applied for targeted gene disruption in vertebrates such as rat, mouse, and zebrafish, and has been widely used to model development and disease (Liu *et al.* 2020b, Wierson *et al.* 2019). In rodent embryos and human cell lines, highly efficient knock-ins, ranging in size from small restriction sites (using DNA oligo as a donor) to medium-sized Cre recombinase and fluorescent protein tags to a large (14 kb) multiple-protein expression cassette, were achieved with MAD7 (Liu *et al.* 2020b).

In cell culture, MAD7 is useful for the targeted integration of a donor vector by microhomology-mediated end joining (MMEJ) or single-strand annealing (SSA) machinery for homology-directed repair into the safe harbor locus AAVS1 in human cells (Wierson *et al.* 2019). Chinese hamster ovary (CHO) cells have been widely used in antibody production. The 4 × nuclear localization signals (NLS) variant of MAD7, rather than the 1 × NLS variant of MAD7, was found to be efficient for generating indel mutations in genome editing using RNP and plasmid-based delivery protocols in CHO cells (Rojek *et al.* 2023). Furthermore, MAD7 has been applied in practical cell line engineering, including glutamine synthetase (GS)-knockout cell lines, which can be used in the GS-mediated gene amplification system (Cockett *et al.* 1990, Noh *et al.* 2018). In addition, targeted integration of a recombinase-mediated cassette exchange (RMCE) landing pad was successfully achieved at the safe harbor site T9 through homologous recombination in CHO cells (Pristovšek *et al.* 2019, Rojek *et al.* 2023). In such cell lines, insertion of a different transgene is easier through DNA exchange reactions with recombinase at the recombinase site of the RMCE landing pad, and isogenic clones with predictable gene expression can be established efficiently (Inniss *et al.* 2017, Kelley 2020, Lee *et al.* 2019a). In the Jurkat T-cell leukemia cell line, one study identified crRNAs that induce indel mutations at a rate of more than 60% at several immune checkpoint receptors, checkpoint phosphatase, and TCR signaling subunit genes with no off-target events (Mohr *et al.* 2023). Moreover, chimeric antigen receptor (CAR) insertions in primary T cells were successfully obtained with the MAD7–crRNA RNP complex at a rate exceeding the standard efficiency of therapeutic transgene virus-free technologies (Mohr *et al.* 2023).

**Fig. 1.**
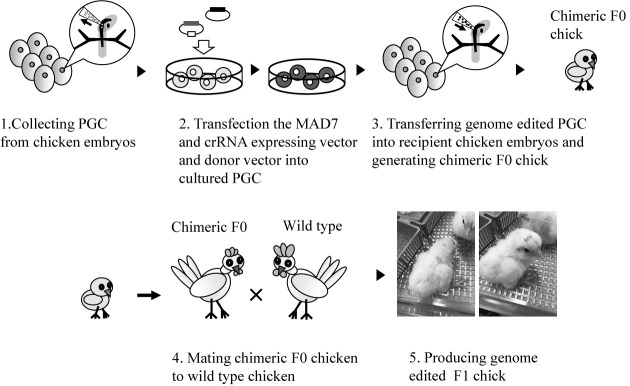
Method of generating a knock-in chicken strain through genome editing. MAD7- and crRNA-expressing vector and donor vector are transfected into PGCs collected from embryos. The PGCs transfected with vectors are collected with a cell sorter, after which the genome-edited PGCs are transferred into the recipient chicken embryos. The chimeric F_0_ chicks are delivered from the recipient and mated with wild-type chickens to generate F_1_ chickens.

Although MAD7 is available for genome editing in various species, as described above, it has not been used in livestock. Our research group focuses on the breeding of livestock to deal with the rapidly growing demand for meat caused by the increasing human population and changing climate. We attempted to establish a genome editing method in chicken. In the case of mammals such as mice and pigs, genome editing is performed after fertilization because fertilized eggs can be obtained relatively easily by in vitro fertilization. However, in the case of chicken, fertilized eggs cannot be used for genome editing because they are in the body of the female (oviduct). Therefore, genome-edited chickens are typically established by performing genome editing at the stage of primordial germ cells (PGCs), which are the source of sperm and eggs, and transplanting the genome-edited PGCs into recipient chickens (Fig. 1). To establish a new recipient chicken line for PGC transplantation in which host germ cells can be removed in a drug-dependent manner, we generated a chicken strain with mCherry and nitroreductase genes inserted into the chicken *Vasa* homolog gene locus through homologous recombination machinery with genome editing using C-terminal NLS-conjugated MAD7 nuclease (Chen *et al.* 2023).

## New genome editing factor ST8

**Fig. 2.**
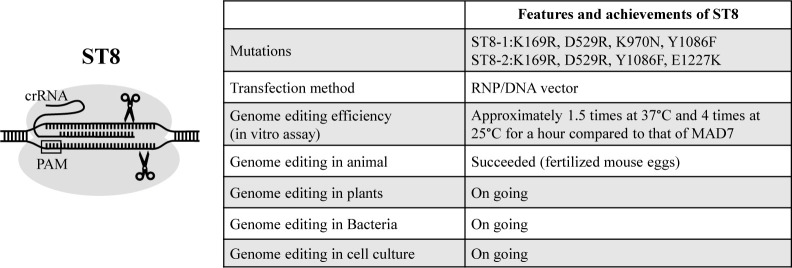
Features and achievements of ST8. ST8-1 and ST8-2 were identified as improved MAD7 variants. The genome editing efficiency of ST8-2 is approximately 1.5 times at 37°C and 4 times at 25°C for 1 h compared with that of MAD7 in vitro cleavage assay. Experiments of genome editing in plants, bacteria and cell culture are currently in progress.

To increase the genome editing efficiency of MAD7, we modified it by inducing mutations and generated a new genome editing factor named ST8. Cleavage activity of ST8.2 harboring K169R, D529R, Y1086F, and E1227K mutations was approximately 1.5 times at 37°C and 4 times at 25°C for 1 h compared with that of MAD7 in vitro cleavage assay (Fig. 2) (Hozumi *et al.* 2023, WO2023145833). ST8 is available for genome editing of fertilized mouse eggs (Fig. 2) (Hozumi *et al.* 2023, WO2023145833). Several improvements can be made to Cas12a, MAD7, and ST8 to optimize the genome editing of plants and crops.

(1) Enhancing genome editing efficiency at low temperature

**Fig. 3.**
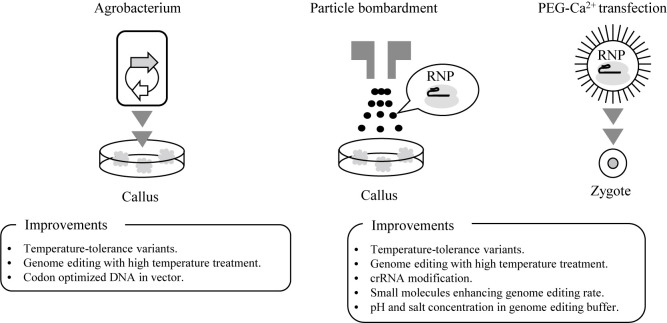
Improvements in genome editing of plants with Cas12a, MAD7, and ST8. Cas12a, MAD7, and ST8 may be used to transfect using Agrobacterium, particle bombardment, and PEG-Ca^2+^ into the callus and zygote of plants. Genome editing in plants can be improved by high-temperature treatment, temperature-tolerant variant, crRNA modification, small molecules, codon-optimized cDNA, and pH and salt concentration in genome editing buffer.

Although LbCas12a is used for genome editing in several plants, there is the limitation in the genome editing of plants that Cas12a orthologs, MAD7 and ST8, are not effective at lower temperatures compared with Cas9. It has been reported that LbCas12a harboring the single mutation D156R provides strong temperature tolerance in the genome editing of *A. thaliana* and tobacco (Huang *et al.* 2021, Schindele and Puchta 2020). It has also been reported that the genome editing efficiency of Cas12a was enhanced using modified crRNA with extension at the 5ʹ end, uridinylate-rich 3ʹ-overhang, ribosyl-2ʹ-O-methylation in the uridinylate-rich 3ʹ-overhang, and five 2ʹ-fluororibose at the 3ʹ termini (Bandyopadhyay *et al.* 2020, Bin Moon *et al.* 2018, Ha *et al.* 2020, Li *et al.* 2017, Park *et al.* 2018). In addition, the small molecules VE-822 and AZD-7762 enhanced the genome editing efficiency with Cas12a (Bandyopadhyay *et al.* 2020, Ma *et al.* 2018). Meanwhile, genome editing under high-temperature conditions resulted in high mutation rates with AsCas12a or LbCas12a in rice, *Arabidopsis*, and maize by *Agrobacterium*-mediated transformation (Malzahn *et al.* 2019). These reports indicate the possibility that these improvements could boost genome editing efficiency in plants and crops with Cas12a, MAD7, and ST8 (Fig. 3).

(2) Optimizing transfection systems

*Agrobacterium*-mediated transformation is a major method in the transformation of a genome editing factor in plants. It exploits the ability of *Agrobacterium* to integrate T-DNA of tumor-inducing (Ti) plasmid into host genomic DNA. Plasmids containing codon-optimized Cas12a, MAD7, and ST8 cDNA and guide RNAs are required for genome editing with this method (Fig. 3). *Agrobacterium*-mediated transformation results in the random integration of the vector fragments into plant genomes. From the perspective of biosafety, it is necessary to obtain lines from which foreign genes have been removed by the genetic separation of sexual generations. However, this is impossible (Liu *et al.* 2022). To avoid importing foreign genes, RNP transfection methods by particle bombardment or polyethylene glycol (PEG)–Ca^2+^ mediation have been developed in the genome editing of plants (Liang *et al.* 2017, Toda *et al.* 2019, 2023). We aim to optimize the conditions of pH and salt concentration in buffer for the genome editing of plants with ST8. We attempted to modify methods to increase genome editing efficiency using ST8 to generate genome-edited plant strains. We expect that ST8 will be available for efficient plant breeding in the near future.

## Author Contribution Statement

S.H. and S.S. wrote the manuscript. Y.C.C. and T.T. contributed to analyzing the chicken data.

## Figures and Tables

**Table 1.** List of crops in genome editing with Cas12a

Plant name	Gene editing system	*Target genes*	Transformation method	Results	Phenotypes	References
Rice	FnCas12a	*OsDL*	Agrobacterium	Mutations	Loss of midrib in the leaf blade, resulting in a drooping leaf phenotype	Endo *et al.* 2016
		*OsALS*, *OsNCED1*, *OsAO1*			n.d.	
Rice	FnCas12a, LbCas12a	*OsCAO1*	Bombardment method	Gene disruption with targeted insertion	Leaf color changed from green to yellow.	Begemann *et al.* 2017
Rice	AsCas12a, LbCas12a	*OsPDS*	Agrobacterium	Mutations	Albino	Tang *et al.* 2017
		*OsROC5*			Curly leaf phenotype	
		*OsDEP1*			n.d.	
Rice	FnCas12a, LbCas12a	*OsPDS*	Agrobacterium	n.d.	Albino	Wang *et al.* 2017
		*OsBEL*, *OsEPSPS*, *OsRLK*		Mutations	n.d.	
Rice	LbCas12a	*OsPDS*	Agrobacterium	Mutations	Albino	Xu *et al.* 2017
		*OsBEL*			n.d.	
Rice	LbCas12a	*OsEPFL9*	Agrobacterium	Mutations	n.d.	Yin *et al.* 2017
Rice	LbCas12a	*OsALS*	Bombardment method	Targeted gene replacement	n.d.	Li *et al.* 2018
Rice	FnCas12a, FnCas12a-RR, FnCas12a-RVR, LbCas12a-RR, LbCas12a-RVR	*OsDEP1*, *OsPDS*, *OsEPFL9*	Protoplast transformation and Agrobacterium	Mutations	n.d.	Zhong *et al.* 2018
Rice	FnCas12a, AsCas12a, LbCas12a	*OsROC5*, *OsDEP1*, *OsPDS*	Agrobacterium and PEG-mediated protoplast transformation	Mutations	n.d.	Malzahn *et al.* 2019
Rice	LbCas12a	*OsDEP1*, *OsRPC5*	Protoplast transformation and Agrobacterium	Mutations	n.d.	Tang *et al.* 2019
Rice	LbCas12a	*OsPDS*, *OsGS3*, *OsALS*, *OsNAL*	Agrobacterium	Mutations	n.d.	Xu *et al.* 2019
Rice	AsCas12a, LbCas12a	*OsPDS*	Bombardment method	Mutations	Albino	Banakar *et al.* 2020
Rice	FnCas12a	*OsDL*, *OsALS*, *OsLCD*, *OsAAO2*, *OsNCED1*	Agrobacterium	Mutations	n.d.	Negishi *et al.* 2020
Rice	LbCas12a	*OsXa13*	Agrobacterium	Mutations	Resistance to bacterial leaf blight	Yu *et al.* 2021
Rice	LbCas12a, LbCas12a-D156R, LbCas12a-RV, LbCas12a-RRV	*OsGA1*, *Os03g52594*, *Os11g20160*, *Os01g09810*, *Os12g12600*, *Os02g54120*, *Os11g19880*, *Os03g52910*	Agrobacterium	Mutations	n.d.	Zhang *et al.* 2023
Maize	LbCas12a	*ZmGL2*	Agrobacterium	Mutations	dull leaf surface retaining water drops	Lee *et al.* 2019b
Maize	LbCas12a	*ZmGL2*	Agrobacterium	Mutations	n.d.	Malzahn *et al.* 2019
Maize	Cpf1	*ZmO2*	Agrobacterium	Mutations	opaque kernels	Gong *et al.* 2021
Wheat	LbCas12a	*GUS*	Agrobacterium	Mutations	n.d.	Liu *et al.* 2020a
Tomato	LbCas12a	*SlAnt1*	Agrobacterium	Gene disruption with targeted insertion	Color of plants turned purple.	van Vu *et al.* 2020
		*SlHKT1;2*		Targeted gene replacement	Salinity tolerance	
Apple	LbCas12a	*MdPDS*	Agrobacterium	Mutations	Albino	Schröpfer and Flachowsky 2021
Dancan fruit	LbCas12a	*CsPDS*, *CsLOBP*	Agrobacterium	Mutations	n.d.	Jia *et al.* 2019
Cotton	LbCas12a	*GhCLA1*	Agrobacterium	Mutations	Albino	Li *et al.* 2019
Tobacco	FnCas12a	*NtSTF1*, *NtPDS*	Agrobacterium	Mutations	n.d.	Endo *et al.* 2016

**Table 2.** Achievements of MAD7 in genome editing

Organisms or Cell lines		Target genes	Results	Delivery form	Phenotypes	References
Plants	Wheat (*Triticum aestivum*)	*TaDEP1*, *TaVRN1*	Indel mutations	Vector	n.d.	Lin *et al.* 2021
	Japonica rice (*Oryza sativa*)	*OsALS*, *OsEPSPS*, *OsNRAMP5*	Indel mutations		n.d.	
Fungi	*Aspergillus nidulans*	*yA*	Stop codon introduction with donor ssDNA or gene disruption with KI in NHEJ-deficient strain	Vector	Conidia color changed from green to yellow.	Vanegas *et al.* 2023
	*Aspergillus niger*	*albA*	Mutation in NHEJ-proficient strain		Conidia color changed from black to white.	
			Stop codon introduction with donor ssDNA or gene disruption with KI in NHEJ-deficient strain			
	*Aspergillus oryzae*	*ku70*	Gene disruption with KI in NHEJ-proficient strain		n.d.	
	*Aspergillus campestris*	*ku70*	Gene disruption with KI in NHEJ-proficient strain		n.d.	
Bacteria	*Bacillus subtilis*	*trpC2*	Stop codon introduction with donor DNA	Vector	Number of colony-forming unit (CPU) was decrease in M9 medium with tryptophan.	Price *et al.* 2020
		*amyE*, *gfpmut3*			α-amylase activity was decreased.	
	*Escherichia coli*	*galK*, *endA*, *fruR*, *relA*, *deoR*, *nupG*	Deletion with donor ssDNA		n.d.	Mund *et al.* 2023
Aminals	Chicken	*Chicken Vasa homolog*	KI of *mCherry* and *nitroreductase* genes	Vector	Cell ablation of PGC with metronidazole	Chen *et al.* 2023
	Mouse (*Mus musculus*)	*Rosa26*	Indel mutations	mRNA	n.d.	Liu *et al.* 2020b
			KI of ssODN donor or expression cassette	mRNA or protein		
	Rat (*Rattus norvegicus*)	*Calb2*	KI of T2A-Cre cassette	Plasmid	n.d.	
	Zebrafish (*Danio rerio*)	*notochord*	Indel mutations	mRNA	loss of the notochord and a shortened tail	Wierson *et al.* 2019
		*cx43.4*			n.d.	
Cell lines	HEK293T	*AAVS1*, *CCR5*, *TRAC*	Indel mutations	Vector	n.d.	Wierson *et al.* 2019
	HCT116	*PPIB*, *DNMT3B*, *NF1*, *STAG2*, *ALK2*, *CACNA1D*, *PPP1R12C*	Indel mutations	Vector or protein	n.d.	Liu *et al.* 2020b
	U2OS	*CBX*	KI of eGFP gene	Vector	n.d.	
	CHO	*Bgn*, *Timp1*	Indel mutations	Vector or protein	n.d.	Rojek *et al.* 2023
		*glutamine synthetase* (*GS*)			GS mutant CHO cell could not survive without L-glutamine supplementation.	
		*T9*	KI of donor plasmid	Vector	n.d.	
	Jurkat	*DNMT1*, *CD247*, *CTLA4*, *LAG3*, *PDCD1*, *PTPN11*, *PTPN6*, *TIGIT*, *TIM3*	Indel mutations	Protein	n.d.	Mohr *et al.* 2023
		*AAVS1*	Insertion of GFP expression cassettes		n.d.	
	Human primary T-cell	*CD247*, *CTLA4*, *LAG3*, *PDCD1*, *PTPN11*, *PTPN6*, *TIGIT*	Indel mutations		n.d.	
		*AAVS1*	Insertion of chimeric antigen receptor (CAR) transgene		n.d.	
